# Posttraining Epinephrine Reverses Memory Deficits Produced by Traumatic Brain Injury in Rats

**DOI:** 10.1155/2016/9151490

**Published:** 2016-04-04

**Authors:** Alejandro Lorón-Sánchez, Meritxell Torras-Garcia, Margalida Coll-Andreu, David Costa-Miserachs, Isabel Portell-Cortés

**Affiliations:** Departament de Psicobiologia i de Metodologia de les Ciències de la Salut, Institut de Neurociències, Universitat Autònoma de Barcelona, Edifici B, 08193 Bellaterra, Barcelona, Spain

## Abstract

The aim of this research is to evaluate whether posttraining systemic epinephrine is able to improve object recognition memory in rats with memory deficits produced by traumatic brain injury. Forty-nine two-month-old naïve male Wistar rats were submitted to surgical procedures to induce traumatic brain injury (TBI) or were sham-operated. Rats were trained in an object recognition task and, immediately after training, received an intraperitoneal injection of distilled water (Sham-Veh and TBI-Veh group) or 0.01 mg/kg epinephrine (TBI-Epi group) or no injection (TBI-0 and Sham-0 groups). Retention was tested 3 h and 24 h after acquisition. The results showed that brain injury produced severe memory deficits and that posttraining administration of epinephrine was able to reverse them. Systemic administration of distilled water also had an enhancing effect, but of a lower magnitude. These data indicate that posttraining epinephrine and, to a lesser extent, vehicle injection reduce memory deficits associated with TBI, probably through induction of a low-to-moderate emotional arousal.

## 1. Introduction

Traumatic brain injury (TBI) is a leading cause of chronic disability in industrialized countries that causes structural damage and functional deficits due to both primary and secondary injury mechanisms [1]. The most common disabilities include sensory and motor deficits, along with cognitive problems, including the spheres of attention, episodic memory, executive functions, working memory, information processing speed, language functions, and visuospatial processing. Among the cognitive deficits, long-term persistence of memory impairment is quite common [2, 3].

Although many treatments have been tested to reverse TBI-induced cognitive deficits, it is still necessary to search for additional therapeutic treatments aimed at reducing cognitive impairment. Many treatments focus on reducing neuronal death by administering neuroprotective agents shortly after trauma [4]. Other treatments are chronically administered to enhance activation of those functional systems affected by TBI [5–8]. It is possible that, along with these treatments, acute treatments administered contingent to a given task could improve learning and memory in a phasic way by affecting the specific cognitive processes triggered by the task, thus extending the treatment strategies for TBI-induced memory deficits. In this sense, enhancement of memory modulating hormones during or shortly after training in a learning task could contribute to cognitive recovery. Posttraining epinephrine is a well-known treatment able to improve memory consolidation of a variety of tasks in healthy animals ([9–12]; for review see [13]), and there are evidences that this effect is higher when memory demands are increased [11] and with healthy animals endowed with lower learning capacities [14, 15]. These evidences support the possibility that posttraining epinephrine could also be effective in reducing memory deficits in animals with brain damage.

Taking into account these considerations, the aim of the current study was to investigate whether the administration of posttraining epinephrine could reverse object recognition memory deficits associated with TBI. Previously, it has been shown that epinephrine is capable of improving memory for this task in healthy animals [11]. In order to examine whether this hormone can also reduce memory deficits after TBI, in the present work the level of difficulty of the task was adjusted to ensure that sham-operated rats had a good recall of the objects, whereas memory deficit was found in TBI animals.

In the present experiment, among the different animal models developed to reproduce the spectrum of pathological changes of human TBI, a controlled cortical impact (CCI) was used. CCI has been widely used in animal research [16] and produces similar deficits of human TBI, such as learning and memory deficits [16, 17], including deficits in object recognition task [18, 19].

## 2. Materials and Methods

We used 49 two-month-old naïve male Wistar rats bred in our laboratory (mean weight 297.9 ± 27.8 g). All rats were housed singly, kept under a 12 h light-dark cycle (lights on at 8:00 a.m.) and in controlled temperature (20–22°C) and humidity (40–70%) conditions. Food and water were provided ad libitum. All procedures were performed in compliance with the directive for care and use of laboratory animals of the European Community Council (86/609/EEC) and of the Autonomous Government of Catalonia (DOGC 2073 10/7/1995).

Starting from the day after being housed, the animals were handled for 5 min on three consecutive days and were randomly assigned to either TBI or sham injury. TBI was produced using a CCI device (Pittsburgh Precision Instruments, Inc., Pittsburgh, USA). Deep anesthesia was induced using 5% isoflurane (Forane®, Abbot Laboratories, Madrid, Spain) in oxygen (2 L/min) in a Plexiglas chamber (20 × 13 × 13 cm) for 7 min and maintained using a nose mask with 2% isoflurane in oxygen (1 L/min). Rats were placed in a stereotaxic frame (David Kopf Instruments, Tujunga, USA) and a midline scalp incision was made followed by a craniotomy (4 mm diameter) in the right hemisphere (+0.45 mm posterior to Bregma and +0.3 mm lateral to the midline). The impactor rod was angled 15° degrees vertically and had an impact tip of 3 mm. The parameters of the impact were velocity of 6 m/s; dwell time of 150 mms; and 2 mm depth from the dura mater surface. At the end of surgery, 0.2 mL buprenorphine (Buprex, Schering-Plough, SA, Madrid, Spain) was administered. Similar surgical procedures were conducted in Sham animals, including anesthesia and craniotomy, but without the cortical impact.

For object recognition procedures, an open box (65.5 cm width × 65.5 cm length × 35 cm height) made of conglomerate covered with brown melamine, enclosed in a sound-attenuating cage (72 cm width × 72 cm length × 157 cm height) made from white melamine, and ventilated by an extractor fan, was used. The illumination on the floor of the box was 30 lux. Four different objects, not known to have any ethological significance for the rats and completely new for the animals, varying in shape, color, and size, were used, three for the object recognition task (an object constructed from Lego, a can, and a plastic hanger) and one for the neophobia test (object constructed from Lego). They were fixed to the floor with adhesive tape to prevent the animals from moving them. To avoid the presence of olfactory cues, the apparatus and objects were thoroughly cleaned with a 70% ethanol solution and dried before the first rat and after each animal. All behavioral sessions were recorded by a video camera mounted above the experimental apparatus. Tapes were analyzed offline by a trained observer who was unaware of the experimental condition of the animals or of which object was familiar with and which was the novel one.

One week after surgery, rats received three sessions of habituation to the experimental apparatus on two consecutive days (two sessions on the first day, 2 h interval, one session on the second day). To determine the emotional reactivity to a novel object, a neophobia test was conducted 2 h after the last habituation. The animals were placed in the box facing away from an object located in the center of the box and were allowed to explore it for 10 min. The time exploring the object was recorded. Throughout the experiment, exploration was defined as directing the nose at the object at a distance of ≤2 cm or touching it with the nose. Turning around or sitting on the object was not considered exploratory behavior. Object recognition training took place the day after the neophobia test. In the training session, two identical objects were placed near the two corners of one side of the cage. The rats were placed in the experimental apparatus, facing the center of the opposite wall, and were allowed to explore for 15 min. Time spent exploring each object was recorded. An exclusion criterion of a minimum of 10 s of total exploration in the training trial was established.

Immediately after training, the animals received either 0.01 mg/kg intraperitoneal (i.p.) epinephrine [(−)epinephrine bitartrate, Sigma Chemical, Madrid, Spain] (Epi group), i.p. distilled water (vehicle; Veh groups), or no injection (0 groups). The dose of epinephrine was chosen according to previous experiments [11]. Thus, the final experimental groups were the following ones: Sham-0, Sham-Veh, TBI-0, TBI-Veh, and TBI-Epi.

Retention was tested 3 h (RT1) and 24 h (RT2) after the training trial. In both sessions, one copy of the familiar object and a novel object were placed in the same location as that of the objects used during the training trial. The novel object used in RT1 was different from the one used in RT2. The position of the objects in the tests and the objects used as novel or familiar were counterbalanced. The rats were placed in the open box and allowed to explore both objects for 5 min, and the time spent exploring each object was recorded. To analyze cognitive performance, a discrimination index was calculated [(time exploring the novel object − time exploring the familiar object) × 100/total time spent on both objects], which made adjusting for any differences in total exploration time possible. As this task is based on the natural tendency of rats to explore novelty, an index significantly higher than zero is considered a good recall of the familiar object, whereas an index close to zero is considered a lack of recall [20].

At the end of the experiment, the animals were killed with an overdose of sodium pentobarbital (Dolethal, 200 mg/kg; Vetoquinol S.A., Madrid, Spain) and perfused transcardially with 4% 0.1 M phosphate-buffered paraformaldehyde (PFA), pH 7.4. Brains were removed and stored in PFA for 3 h at −4°C and subsequently rinsed three times (20 min delay) in phosphate buffer at 4°C and were kept 48 h in 30% buffered sucrose at 4°C for cryoprotection. The tissue was then stored at −80°C until being sectioned (40 *μ*m) on a cryostat (Shandon Cryotome FSE, Thermo Electron Corporation, Madison, USA). Throughout the extent of the brain where a lesion cavity was visually apparent, one in ten sections was taken for subsequent staining in cresyl violet and digitalized with a scanner (HP Scanjet G4050). Using the image analysis software ImageJ 1.45 s, digital images were calibrated and the area of the lesion for each slice was measured. To calculate the volume of the lesion, the area of the lesion cavity in each slice was multiplied by 0.04 mm (slices width) and by 10 (number of sections until the next slice analyzed).

SPSS 19.0 (Chicago, USA) was used for data analyses. Analysis of variance (ANOVA) was applied considering group (Sham-0, Sham-Veh, TBI-0, TBI-Veh, and TBI-Epi) as the qualitative independent variable and total exploration times in the neophobia, training, and retention tests as quantitative dependent variables. To analyze retention, repeated measures analyses of variance were used considering group as the independent variable and the discrimination index at RT1 and RT2 as the dependent variables. Bonferroni post hoc tests were conducted when necessary.

One-sample *t*-tests were used to determine whether the discrimination index was different from zero (chance level). Differences in lesion volume were analyzed with an ANOVA considering group (TBI-0, TBI-Veh, and TBI-Epi) as the independent variable and lesion volume as the dependent variable. Statistical significance was set at the level of *P* ≤ 0.05.

One rat in the Sham-Veh group showing a total exploration time < 10 s on acquisition was excluded from the analyses. Boxplot identified six subjects (Sham-0 = 1, Sham-Veh = 2, TBI-Veh = 1, and TBI-Epi = 2) with outlier values in RT1 or RT2, which were excluded from the analyses. Thus, the total number of rats per group was as follows: Sham-0 (*n* = 9), Sham-Veh (*n* = 7), TBI-0 (*n* = 9), TBI-Veh (*n* = 10), and TBI-Epi (*n* = 7).

## 3. Results

**Figure 1:**
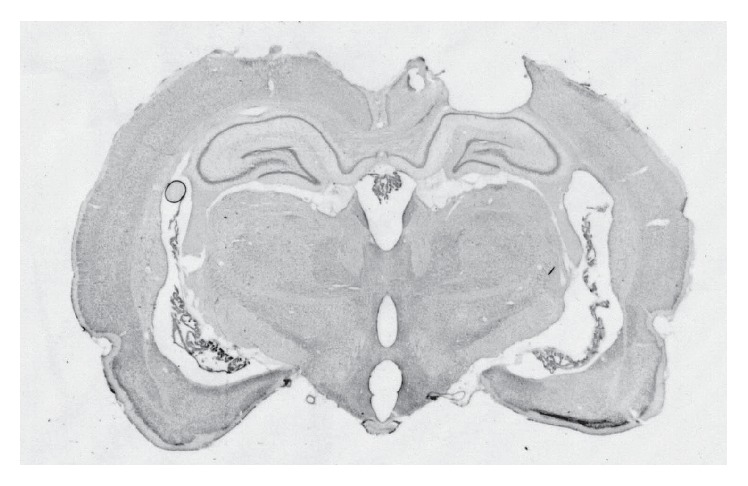
Representative lesion induced by CCI.

Figure 1 shows a coronal section of the brain in one representative TBI subject. The mean lesion volume in mm^3^ (±SD) for each group was TBI-0 (5.2 ± 3), TBI-Veh (7.1 ± 4), and TBI-Epi (5.7 ± 3.3). No significant differences were found between groups.

No significant differences were found among groups in total exploration time during the neophobia, training, or retention sessions (see Table 1).

**Figure 2:**
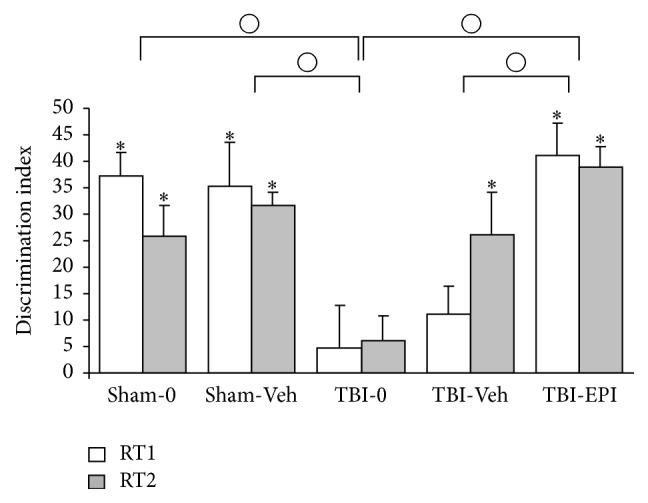
Mean value (+SEM) of the discrimination index at 3 h and 24 h retention tests. *∗*: statistically significant differences compared to zero; ○: statistically significant differences between groups.

*t*-tests showed that the discrimination index in RT1 was significantly different from zero (indicating a significant recall) in Sham-0 (*t*_8_ = 8.42, *P* < 0.001), Sham-Veh (*t*_6_ = 4.24, *P* = 0.005), and TBI-Epi (*t*_6_ = 6.72, *P* = 0.001) groups and did not differ from 0 (indicating lack of recall) in TBI-0 and TBI-Veh groups. In RT2, the discrimination index was significantly different from zero in all groups except for TBI-0 group (Sham-0: *t*_8_ = 4.5, *P* = 0.002; Sham-Veh: *t*_6_ = 13.2, *P* < 0.001; TBI-Veh: *t*_9_ = 3.25, *P* = 0.010; and TBI-Epi: *t*_6_ = 10, *P* < 0.001). Therefore, whereas TBI-0 did not remember the familiar object on RT1 and RT2 and TBI-Veh on RT1, TBI-Epi and Sham groups remember it on both retention sessions (Figure 2).

Mixed analyses of variance showed that the main factor group was significant (*F*_4,37_ = 10.56, *P* < 0.001), while neither* session* nor* group* ×* session* interaction was significant. Bonferroni post hoc tests showed that the discrimination index of TBI-0 group was significantly lower than that of Sham-0 (*P* = 0.001), Sham-Veh (*P* = 0.001), and TBI-Epi (*P* < 0.001) groups, but not lower than that of TBI-Veh group. The same analyses showed that TBI-Veh was significantly lower than that of TBI-Epi (*P* = 0.01). No other differences were detected (Figure 2).

## 4. Discussion

The main results from this study were as follows: (1) TBI produced significant impairment of object recognition memory, (2) posttraining i.p. epinephrine was able to reverse this memory deficit, and (3) systemic administration of distilled water also had a positive effect, but of smaller magnitude.

Our data demonstrates that TBI produces object recognition memory deficits at the two times tested (3 and 24 h). Specifically, while in nonlesioned rats (Sham-0) the discrimination index was significantly different from zero at both delays, the discrimination index of TBI-0 group did not differ from zero in any of the retention sessions. Moreover, performance of TBI-0 group was significantly lower than that of Sham groups. This deficit is consistent with previous results using object recognition memory in rodents [18, 19, 21–26] and also with other studies with different behavioral procedures in both humans and animals, thus showing that anterograde amnesia is a common sequel of TBI [3, 17]. The group differences in behavioral outcome were not likely due to differences in anxiety when faced with a new object or in exploration times during acquisition or retention sessions, as no differences between groups were observed when these variables were analyzed. Spontaneous object preference can also be ruled out because new and old objects were counterbalanced. However, since TBI was present before the beginning of the behavioral procedures, it is not possible to establish whether the observed effects are due to differences in attending or encoding information during training or to deficits in storage or retrieval information.

Posttraining epinephrine reversed these memory deficits. Injured rats with posttraining epinephrine (TBI-Epi group) spent significantly more time exploring the novel than the familiar object in both retention sessions, showing good recall of the task on both sessions. Moreover, their level of performance was significantly higher than that of lesioned rats (TBI-0 and TBI-Veh) and similar to that of nonlesioned rats (Sham-0 and Sham-Veh). That is, the amount of enhancement of posttraining epinephrine was enough to bring lesioned rats' memory performance to the level of nonlesioned rats.

What may have been the mechanisms mediating the benefits of epinephrine on performance in memory tests? It is highly unlikely that the effects found might be attributed to a neuroprotective action, since epinephrine was administered only once, in a posttraining contingent basis (immediately after the acquisition session) and 10 days after injury (and thus, not in the acute postinjury period). In concordance with this, no differences in the size of the lesion cavity were found between the different TBI groups. The fact that epinephrine was administered after the acquisition session also rules out the possibility that the improvement effect was due to epinephrine-induced effects on attention, motivational, motor, or sensory processes during training. We cannot rule out that the effect on 3 h retention could be due to a proactive effect on retrieval, since the fact that some effect induced by this treatment could persist along this relatively short delay between injection and retention cannot be disregarded. Nevertheless, this possibility seems unlikely since it has been reported that posttraining epinephrine improved object recognition memory when tested 96 h, but not 1.5 h, after treatment [27]. In contrast, the effect of posttraining epinephrine is consistent with multiple data in healthy animals indicating that this treatment enhances memory by acting on memory consolidation mechanisms [13]. While there is substantial evidence that epinephrine modulates memory for highly arousing aversively motivated task, the present results confirm previous reports in nonlesioned rats showing that posttraining epinephrine is also able to improve memory for tasks with a very low emotional component such as object recognition [11, 27].

As epinephrine does not readily cross the blood-brain barrier [28] its effects on memory consolidation appear to be initiated on peripheral actions. There are two major views regarding the mechanisms by which epinephrine can modulate memory consolidation. One is based on the idea that epinephrine activates the beta-adrenoceptors located on vagal afferents that project to the nucleus of the solitary tract in the brain stem that sends noradrenergic projections to forebrain regions including the amygdala. Another view suggests that the effects of posttraining epinephrine would be mediated by the glucose released into the blood by the activation of hepatic adrenoreceptors [13, 29]. While both vagal- and glucose-mediated pathways have been explored to decrease cognitive deficits after brain injury [30, 31], to our knowledge this is the first time that a single and contingent posttraining epinephrine dose has been shown to revert memory deficit seen after a long postinjury delay (10 days).

Intraperitoneal administration of distilled water also exerted a positive, but less powerful, effect on cognition. Rats of the TBI-Veh group were able to remember the familiar object, but only on the second retention session, and showed a level of performance lower than TBI-Epi group but intermediate between Sham rats and TBI nontreated rats. In this sense, performance of rats of TBI-Veh group was significantly lower than that of TBI-Epi group but did not significantly differ from Sham-0, Sham-Veh, and TBI-0 groups. Although we do not have direct measures of that, the present results could suggest that the injection of distilled water induced some degree of emotional arousal that was able to enhance memory modulation, in a similar way than posttraining epinephrine. Since this effect of posttraining injections on memory has not been found in nonlesioned rats (Sham-Veh) or in previous experiments using similar handling procedures and memory task [11], the present results might indicate that even low levels of emotional arousal can enhance performance in memory tasks in animals that present a clear memory deficit, such as after TBI, at least at the postinjury time point used here (10 days).

Our results suggest that an acute contingent posttraining treatment could be a potential way to alleviate memory deficits associated with TBI. It is important to note that posttraining injections of epinephrine would not be the best choice for patients due to several clinical contraindications (e.g., sulfate sensitivity, diagnosis with closed-angle glaucoma, and altered blood pressure) and interactions with several drugs (e.g., tricyclic antidepressants, halogenated anesthetics, and beta blockers) [32]. However other therapeutic strategies could be used in humans to induce an arousal level similar to that produced with posttraining administration of epinephrine. There is evidence that the induction of a moderate degree of muscle tension, which increases arousal and causes a physiological response similar to that obtained with peripheral administration of posttraining epinephrine, is also capable of positively modulating memory processes in humans [33, 34] and even of bringing memory performance of older adults to the level of unmodulated younger adults [35]. This kind of treatment could be easily and noninvasively self-administered by the patient on demand, for example, by squeezing a hand dynamometer [33, 34] or a sand-filled ball [35].

## 5. Conclusions

This study shows that TBI produced significant impairment of object recognition memory and that posttraining epinephrine was able to reverse this memory deficit. Posttraining administration of vehicle had also a positive effect, but of smaller magnitude. These results raise the possibility that administering acute treatments contingently to a given task and aimed at improving the specific cognitive processes triggered by it could constitute a complementary approach to therapeutic strategies targeted to reduce the alterations in brain function associated with brain damage.

## Figures and Tables

**Table 1:** Mean values (SEM) of the total exploration times (s) in the neophobia, acquisition, and retention sessions for each experimental group.

	Sham-0	Sham-Veh	TBI-0	TBI-Veh	TBI-Epi
Neophobia	88.1 (16)	99 (21.2)	71.7 (22.7)	121.3 (18.2)	108.3 (18.5)
Acquisition	111.4 (14.5)	146.3 (19.7)	96 (21.8)	145.2 (10.6)	122 (17.4)
3 h retention	48.2 (6.9)	60 (6.8)	52 (5.4)	57 (5.4)	57.7 (9.4)
24 h retention	57.4 (8.7)	47 (4.9)	43.9 (6.8)	43.7 (4)	40.6 (4.5)
